# Virtual Reality in Prevention and Treatment of Substance‐Related Disorders: A Systematic Review of Randomized Controlled Trials

**DOI:** 10.1002/cpp.70144

**Published:** 2025-08-22

**Authors:** Renata Glavak‐Tkalić, Mara Šimunović, Katarina Perić Pavišić, Josip Razum, Desirèe Colombo

**Affiliations:** ^1^ Ivo Pilar Institute of Social Sciences Zagreb Croatia; ^2^ School of Health Sciences, Faculty of Psychology University of Iceland Reykjavik Iceland; ^3^ Polibienestar Institute, Department of Personality, Evaluation and Psychological Treatments University of Valencia Valencia Spain

**Keywords:** addiction, prevention, randomized controlled trials, substance abuse, substance‐related disorders, treatment, virtual reality

## Abstract

**Background:**

Substance abuse (SA) imposes a significant global health burden, demanding innovative and accessible interventions. Virtual reality (VR) offers a promising approach, providing engaging and personalized treatment experiences. However, rigorous evidence from randomized controlled trials (RCTs) on VR's efficacy in the treatment and prevention of SA remains limited. This systematic review aimed to characterize VR interventions for substance‐related disorders and evaluate their effectiveness.

**Methods:**

To conduct this review, two researchers independently performed a comprehensive literature search across four databases using the Preferred Reporting Items for Systematic Reviews and Meta‐Analyses (PRISMA) guidelines.

**Results:**

Twenty RCTs met the inclusion criteria, focusing on alcohol, nicotine and illicit drug use. These studies utilized diverse VR modalities, most frequently exposure therapy (*n* = 10) and cognitive‐behavioural therapy (*n* = 5), followed by approach bias modification, skills training, cognitive rehabilitation, counterconditioning and psychoeducation. Interventions varied in level of immersion and interactivity. Although the evidence was mixed, 17 studies demonstrated positive effects on at least one outcome variable. Most studies focused on proximal outcomes (e.g., craving), which frequently showed improvement. Clinically meaningful outcomes (e.g., substance use reduction and abstinence) were less frequently assessed, with seven of 10 studies reporting improvement.

**Conclusions:**

VR shows promise in addressing substance‐related disorders, particularly for alcohol and nicotine. However, substantial heterogeneity in VR interventions highlights the need for further research to standardize methodologies, optimize treatment parameters and explore the underlying working mechanisms of VR interventions. Additional research is also needed to assess VR's application to illicit drug use.

## Summary

- Virtual reality (VR)–based interventions, particularly those that integrate cue exposure therapy and cognitive behavioural therapy, show significant promise in reducing cravings and improving abstinence among individuals using alcohol and nicotine.
- VR intervention and prevention programmes have positively impacted attitudes, intentions, cognitive function and physiological responses in substance users, indicating a broader therapeutic potential that extends beyond simply addressing addiction symptoms.
- The considerable variability among VR interventions emphasizes the need for greater standardization in methodologies, treatment parameters and outcome measures.
- Additional research is necessary to evaluate the applicability and efficacy of VR in the prevention and treatment of illicit drug use.

## 1 Introduction

Substance abuse (SA) is a global threat that poses a significant burden on public health, contributing to a wide range of health issues, social challenges and economic costs worldwide. In 2020, an estimated 22.3% of the world's population used tobacco (World Health Organization 2021). In 2022, 5.6% of people aged 15–64 years used psychoactive drugs globally (United Nations Office on Drugs and Crime 2024). An estimated 7% of the global population aged 15 years and older live with alcohol use disorders (AUDs), whereas 3.7% live with alcohol dependence (United Nations Office on Drugs and Crime 2024). Tobacco kills more than 8 million people annually, including those affected by second‐hand smoke (World Health Organization 2021). In 2019, alcohol consumption was attributable to 2.6 million deaths, whereas psychoactive drug use accounted for 0.6 million deaths (World Health Organization 2024). Despite the high prevalence of substance use disorders (SUDs), only a small fraction of individuals receive treatment. In 2022, only about 1 in 11 people with drug use disorders received treatment globally (United Nations Office on Drugs and Crime 2024), and globally, one in six people with AUD received treatment (Mekonen et al. 2021). Those individuals who are involved in treatment often show a chronic, relapsing course and struggle to maintain long‐term abstinence (Cheron and Kerchove d'Exaerde 2021).

The impact of SA on health and development is acknowledged in the 2030 Agenda for Sustainable Development, particularly through Sustainable Development Goal 3.5, which emphasizes the need for enhanced prevention of SA and improved treatment of SUDs (World Health Organization 2024). SUDs are the most commonly encountered disorders in behavioural health care and a leading preventable cause of death in the Western world (Miller et al. 2019). A variety of interventions are available for the treatment of SUDs, both inpatient and outpatient, which include pharmacological and psychosocial approaches, depending on the specific substance and the severity of SUD. Psychosocial interventions include counselling, motivational interviewing, cognitive behavioural therapy (CBT), group therapy, family therapy, case management and relapse prevention (European Union Drugs Agency 2025). However, patients are often reluctant to seek care because of stigma and the limited availability of specialized treatment (Kreek et al. 2019; Miller et al. 2019). Moreover, addiction is recognized as a chronic, progressive and relapsing disorder (National Institute on Drug Abuse 2019), and the efficacy of treatment is often questioned because of the fact that most affected individuals require multiple treatment episodes over several years to achieve recovery (Beaulieu et al. 2021; Dennis and Scott 2007). This is further complicated by high drop‐out rates (Lappan et al. 2020) and high relapse rates (Barati et al. 2021). A meta‐analysis of in‐person SUD treatment studies by Lappan et al. (2020) reported an average dropout rate of 30.4% (95% CI: 27.2–33.8), with substantial heterogeneity. Reported dropout rates ranged from 0% to 100%, depending on the population, type of substance used and characteristics of treatment. A significant association was found between dropout rates and an increased risk of relapse (Brorson et al. 2013). Preventing or reducing the frequency and severity of relapses is one of the fundamental aims of addiction treatment (Hendershot et al. 2011). Equally important is recognizing and addressing lapses—temporary brief episodes of returning to substance use following a period of abstinence—as they may serve as early warning signs and critical intervention points to prevent full relapse and support sustained recovery (Brown 2025). To minimize relapse rates in addiction treatment, several evidence‐based tools and strategies have been developed, with CBT and motivational interviewing among the most commonly used approaches. CBT has proven to be efficient in relapse prevention, both as a monotherapy and in combination with other treatment strategies, while incorporating various treatment elements, including cognitive and motivational as well as skills‐building interventions (McHugh et al. 2010; Parks et al. 2004). Motivational interviewing has also been shown to be effective in reducing relapse rates (Schwenker et al. 2023), especially when combined with other therapeutic approaches such as CBT and mindfulness‐based interventions (Memória et al. 2025).

With advancements in technology, new possibilities for the prevention and treatment of mental health disorders are becoming increasingly accessible. Emerging digital tools, such as mobile applications that deliver evidence‐based interventions (Staiger et al. 2020), reinforcement learning algorithms for personalized interventions (Ghosh et al. 2024) and VR‐based therapies like cue exposure treatment (e.g., Chen et al. 2018; Malbos et al. 2023), may help address difficulties being present in traditional interventions. Increasing access to effective treatment for individuals already affected by SUDs remains crucial to supporting long‐term recovery. Virtual reality (VR) technology, which, as concluded by Lebiecka et al. (2021) in their narrative review, shows promising effects in the treatment of SUDs. The first developed virtual environments were primarily displayed on flat screens, offering limited immersion. However, the development of head‐mounted displays (HMDs) enabled users to perceive depth and move within the environment, creating a compelling illusion of presence in an alternate reality (Freeman et al. 2017) and bringing virtual experiences close to real‐life perception (Baños et al. 2004). Additionally, the rise of low‐cost 360° stereoscopic video cameras and open‐source VR tools has expanded possibilities for clinical use, enabling the development of virtual environments that, despite lacking user interaction or movement, still provide a powerful sense of presence and immersion. In the field of SUD treatment, VR techniques provide an alternative to traditional treatment methods, as they allow individuals to confront substance‐related cues in a controlled setting, potentially leading to better therapeutic outcomes. VR in therapy can personalize treatment by allowing tailored interventions that address specific triggers and cravings for different substances (Mazza et al. 2021), such as simulating real‐life scenarios using cues that lead to these cravings (Ghiţă et al. 2019; Freeman et al. 2017; Segawa et al. 2020).

The integration of VR in the prevention of SUDs allows tailoring experiences to meet the needs of diverse populations. For example, adolescents have reported positive experiences with VR scenarios that exposed them to peer pressure and the consequences of SA (Prediger et al. 2021). VR, as well as other digital interventions (e.g., mobile‐based and game‐based), has the advantage of being perceived as less stigmatizing and more engaging for youth, which can enhance its effectiveness (Monarque et al. 2023).

Previous systematic reviews of VR‐based interventions for SUDs conclude that they show promising effects in the treatment of disorders related to various substances, such as alcohol, nicotine, cannabis, cocaine and methamphetamine (Lebiecka et al. 2021; Tatnell et al. 2022; Trahan et al. 2019; Segawa et al. 2020; Samora et al. 2024), as well as behavioural addictions, including internet gaming disorder and gambling disorder (Segawa et al. 2020). However, these systematic reviews emphasize the need for further scientific evidence (Lebiecka et al. 2021), as they report heterogeneous results (Nègre et al. 2023; Segawa et al. 2020). They also highlight significant methodological issues, such as small sample sizes (Tsamitros et al. 2021), lacking control groups (Tatnell et al. 2022), a high risk of bias in the selected research (Trahan et al. 2019) and a lack of data on the long‐term effects of VR treatment (Tsamitros et al. 2021). Furthermore, only one systematic review (Tatnell et al. 2022) included exclusively randomized controlled trials; however, it focused on smoking, as no studies on the effectiveness of VR on alcohol consumption met the inclusion criteria, and illicit drug use was not included in the review. Furthermore, we found no systematic reviews that focused on VR interventions in both the treatment and prevention of SA. Therefore, considering the limitations of existing systematic reviews and the gaps in the scientific literature, there is a need for a systematic review specifically examining the effectiveness of VR interventions in the treatment and prevention of SA, focusing exclusively on randomized control trials. This systematic literature review has two objectives: (1) to describe different VR interventions that were used to treat or prevent substance (ab)use and (2) to describe the effectiveness of these interventions and their components (i.e., working mechanisms).

## 2 Methods

### 2.1 Search Strategy

The search process was carried out following the guidelines of the Preferred Reporting Items for Systematic Reviews and Meta‐Analyses (PRISMA statement; Page et al. 2021). The systematic literature search was conducted in the Web of Science, PubMed, PsycInfo and Google Scholar databases. The following search terms and logic were used in each database: (‘virtual reality’) AND (intervention OR randomized controlled trial OR evaluation* OR trial* OR campaign* OR program* OR prevention OR ‘pilot study’) AND (‘psychoactive substances’ OR ‘alcohol’ OR ‘binge drinking’ OR ‘heavy episodic drinking’ OR ‘intoxication’ OR ‘illicit drugs’ OR ‘recreational drugs’ OR ‘psychostimulants’ OR ‘stimulant type drugs’ OR ‘MDMA’ OR ‘ecstasy’ OR ‘LSD’ OR ‘hallucinogens’ OR ‘psychedelics’ OR ‘amphetamines’ OR ‘methamphetamines’ OR ‘cannabis’ OR ‘cocaine’ OR ‘new psychoactive substances’ OR ‘novel psychoactive substances’ OR ‘legal highs’ OR ‘ketamine’ OR ‘GHB’ OR ‘GBL’ OR ‘nitrous oxide’ OR ‘synthetic cannabis’ OR ‘synthetic stimulants’ OR ‘synthetic cathinones’ OR ‘medications’ OR ‘prescription drugs’ OR ‘polysubstance use’). Searches were conducted using the title, abstract and keywords of articles to ensure comprehensive coverage.

Two researchers independently conducted the screening process, which was carried out in two phases. In the first phase, the researchers screened all articles retrieved through keyword searches by evaluating their titles and abstracts. When necessary, brief reviews of the full texts were conducted. Each researcher was assigned two of the four databases, which they independently screened: Web of Science, PsycINFO, PubMed and Google Scholar. After completing the initial screening, each of the two researchers cross‐checked the pool of articles screened by the other and reviewed the selected articles. In cases of disagreement, three other authors were consulted, and a unanimous decision was reached.

### 2.2 Inclusion and Exclusion Criteria

For this systematic review, studies were considered eligible if they met the following criteria: (1) They were peer‐reviewed and published in English up to May 2024, which was the time of our data search; (2) they employed VR technology, including augmented reality and mixed reality, as part of an intervention or prevention programme; (3) they provided primary outcomes related to substance (ab)use and addiction prevention or treatment (e.g., craving and abstinence); and (4) they were randomized controlled trials. Studies were excluded if they (1) targeted populations with specific comorbid conditions (e.g., mental health disorders or cognitive impairments); (2) involved interventions or programmes exclusively focused on nonsubstance‐related issues (e.g., general well‐being or lifestyle changes, gaming or gambling disorder) that do not directly address substance (ab)use or addiction; (3) did not provide clear primary outcome measures related to substance (ab)use and addiction prevention or treatment; and (4) were theoretical articles, policy papers or research protocols.

### 2.3 Data Extraction

Two authors independently extracted data from the included studies. Any discrepancies in the extracted information were resolved through discussion until consensus was achieved. The extracted data included the following details: (1) publication details: author(s), year of publication, country of study and years during which data were collected; (2) study objectives: whether the focus was on intervention or prevention; (3) inclusion and exclusion criteria; (4) sample characteristics: age, gender and population (e.g., illicit drug users or heavy social drinkers); (5) sample size: number of participants in the control and experimental groups; (6) type of psychoactive substance targeted (e.g., alcohol, tobacco and illicit drugs); (7) control group type (e.g., treatment as usual and CBT); (8) outcome measures (e.g., smoking abstinence and relapse rates); (9) type of intervention (e.g., standalone VR or combined with another approach); and (10) description of the VR intervention, including duration, number of sessions and specific activities or procedures involved.

### 2.4 Study Selection

In the first phase of the search protocol, a total of 791 were identified. The WOS database contained 321 articles; PubMed, 209; PsychInfo, 161; and Google Scholar, 100 articles (the search was limited to the first 100 articles). Articles were merged into the database, and 237 duplicates were deleted, whereas 554 articles were screened by title and abstracts, resulting in the exclusion of 380 articles that were not relevant to this review, and six articles could not be retrieved, leaving a total of 168 articles eligible for review. In the second phase, full‐text analysis was conducted. Full texts were reviewed independently by the same two authors who conducted abstract screening. Each author read one half of the full texts and decided which articles to exclude and include. Afterwards, each author cross‐checked the pool of articles from the other author and agreed with these choices. In case of disagreements, the other three authors were consulted, and a unanimous decision was reached. We excluded 30 studies because there was no RCT, 22 studies because they did not include VR, 69 studies because they did not include interventions, 16 studies because addiction was not an outcome and 10 studies because they were only the study protocol, with no results presented. One article was excluded because only the extended abstract, not the full article, was published in English. Finally, 20 articles were included in this review.

**FIGURE 1:**
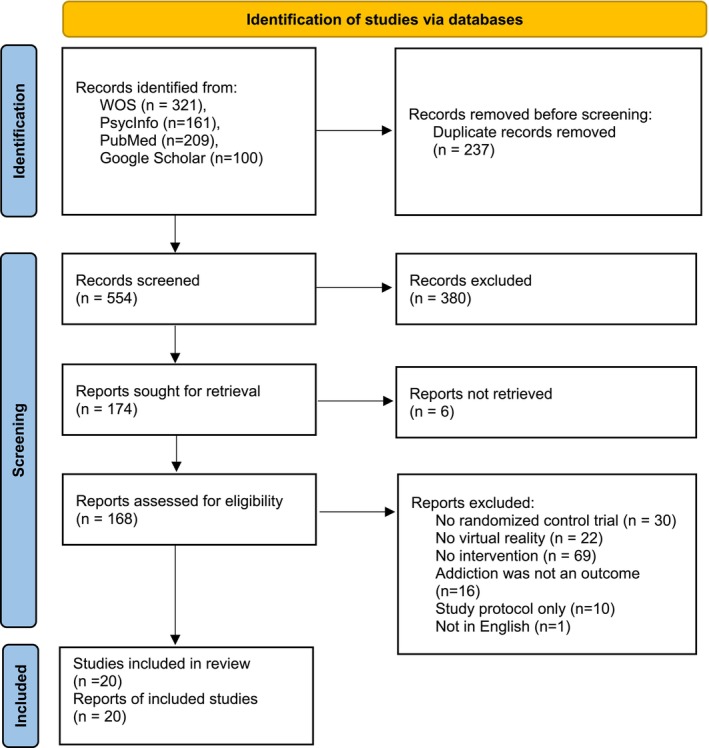
PRISMA flow diagram.

The search process was carried out following the guidelines of the PRISMA statement (Page et al. 2021), and it is presented in the PRISMA flow diagram (Figure 1).

### 2.5 Risk of Bias Assessment

To evaluate the risk of bias in the studies included in our systematic review, we used the revised Cochrane Risk of Bias tool for randomized trials (RoB2; Sterne et al. 2019). The tool assesses five domains: (1) the randomization process (D1), (2) deviations from the intended interventions (D2), (3) missing outcome data (D3), (4) measurement of the outcome (D4) and (5) selection of the reported result (D5). Each domain, as well as the overall bias, was rated as low risk, some concerns or high risk. The results are presented in Table 2.

## 3 Results

Of the 20 articles included in the analysis, in eight articles, VR was implemented to reduce alcohol use and craving for alcohol (Figueras‐Puigderrajols et al. 2020; Gamito et al. 2021; Guldager et al. 2022; Hernández‐Serrano et al. 2020; Kim and Lee 2019; Ma 2020; Thaysen‐Petersen et al. 2024; Zhang et al. 2023), eight articles focused on smoking cessation or reduction in smoking cigarettes (Bordnick et al. 2012; Culbertson et al. 2012; Girard et al. 2009; Goldenhersch et al. 2020; Machulska et al. 2021; Malbos et al. 2018, 2023; Pericot‐Valverde et al. 2019), and one article focused on awareness of the harm of e‐cigarettes (Weser et al. 2021). One article centred on cognitive assessments of ketamine users (Man 2018), and one on the reduction of craving in METH users (Wang et al. 2019). One article focused on opioid overdose knowledge and opioid overdose attitudes (Herbert et al. 2020). Details of the studies are presented in Table 1.

**TABLE 1:** Studies included in the review.

Study	Country	Objectives (I or P)	Participants	N (E, C)	Age (M, SD)	Type of intervention	Immersion/interaction^a^	Control	Intervention description	Outcomes	Findings
	Alcohol										
Kim and Lee (2019)	Republic of Korea	I	Heavy social drinkers	28 (14, 14)	22.36 (2.31); 22.79 (2.89)	VAAAT	Immersion: no Interaction: yes	Sham training	VAAAT training in 3 sessions over 3 days within 2–3 weeks (at the interval of 4–5 days)	1. Implicit approach tendencies towards alcohol 2. Explicit cravings for alcohol	The intervention group showed a decrease in implicit approach tendencies towards alcohol, but not in explicit craving for alcohol. In contrast, the control group showed an increase in both.
Hernández‐Serrano et al. (2020)	Spain	I	Patients with alcohol use disorder	42 (15, 27)	54.60 (7.71)	TAU + VR‐CET	Immersion: yes Interaction: yes	TAU	Eight sessions (2 assessment sessions + 6 VR‐CET sessions using ALCO‐VR software). Each VR‐CET session lasted 50 min	1. Alcohol consumption, drinking behaviours and alcohol‐related problems 2. Alcohol craving 3. Attentional bias for alcohol‐related content 4. State anxiety	The intervention group showed greater changes in improvement in the levels of alcohol craving than the control group. Intragroup changes in alcohol craving from pre‐ to post‐treatment were significant in the intervention group but not within the control group.
Ma (2020)	USA	P	College students	107 (55, 52)	19.06 (n/a)	VR	Immersion: yes Interaction: no	360‐video on iPad	A 360‐video named ‘Decisions: a 360 virtual reality drunk driving experience’ (4 min and 41 s long), using a head‐mounted display	1. Attitudes towards alternatives to drinking and driving 2. Behavioural intentions to engage in drinking and driving 3. Perceived similarity to the protagonist	The immersive story led to lower intentions to drink and drive only among female participants when viewed through a VR headset (vs. iPads).
Figueras‐Puigderrajols et al. (2020)	Spain	I	Patients with alcohol use disorder	38 (12, 16)	53.82 (7.93)	VR‐CET	Immersion: yes Interaction: no	TAU	Eight sessions (2 assessment sessions + 6 VR‐CET sessions using ALCO‐VR software). Each VR‐CET session lasted for 1 h.	1. Alcohol consumption, drinking behaviours and alcohol‐related problems 2. Alcohol craving 3. Attentional bias for alcohol‐related content 4. State anxiety	Although group differences did not reach significance, the decrease in the measures was more evident in the experimental group.
Gamito et al. (2021)	Portugal	I	Individuals with alcohol use disorder undergoing residential treatment	40 (22, 18)	44.83 (12.04)	TAU + VR‐CBT	Immersion: no Interaction: yes	TAU	VR intervention plus TAU during participants' second and third weeks of hospitalization. This intervention consisted of 10 sessions, lasting 30–40 min, twice a week over 5 weeks.	1. Global cognition 2. Executive functions 3. Memory 4. Attention 5. Cognitive flexibility	Results indicated better cognitive and executive functioning in the experimental group than in the control group.
Guldager et al. (2022)	Denmark	P	Students	378 (183, 195)	15.66 (0.76)	VR	Immersion: yes Interaction: yes	Active: control game ‘Oculus Quest—First Steps’	One session included a gameplay introduction (45 min), gameplay (15 min), and structured reflection of the experiences.	1. Drinking refusal self‐efficacy 2. Drug refusal skills 3. Knowledge/awareness of blood alcohol concentration 4. Communication skills 5. Social support willingness 6. Susceptibility to peer pressure 7. Outcome expectations	There is no significant difference between the intervention and control groups.
Zhang et al. (2023)	China	I	Alcohol dependent according to the ICD‐10 criteria	44 (23, 21)	34.30 (5.23)	TAU + VR‐CET	Immersion: yes Interaction: no	TAU	VR‐CET video in 8 sessions, 3 sessions per week (1 day apart) for 8 min	1. Subjective craving 2. Heart rate, skin conductance and respiratory signals	The changes in subjective craving and heart rate in the intervention group were significantly lower than in the control group. In contrast, the changes in skin conductance and respiration between the intervention and control groups were not significantly different.
Thaysen‐Petersen et al. (2024)	Denmark	I	Treatment‐seeking AUD‐diagnosed individuals	9 (5, 4)	48.33 (13.72)	VR‐CBT	Immersion: yes Interaction: no	CBT	VR‐assisted CBT sessions once weekly for 3 weeks; each session lasted 45–60 min. Patients were exposed to a VR high‐risk restaurant scene, which was the basis for cognitive analysis during or after the VR exposure.	1. Feasibility measured through: drop‐out rates, VR‐induced reactions (thoughts, emotions, psychological reactions incl. cravings) and simulator sickness 2. Preliminary efficacy: percentage change from inclusion to 1‐ and 4‐week FU in total alcohol consumption	Patients receiving VR‐CBT had more reduction in alcohol consumption and craving than patients receiving conventional CBT at 1‐week and 1‐month follow‐up.
	Nicotine										
Girard et al. (2009)	Canada (Quebec)	I	People smoking at least 10 cigarettes per day during the last year	91 (46, 45)	44 (11)	Psychosocial support + VR (cigarette crush game)	Immersion: yes Interaction: yes	Psychosocial support + VR (balls crush game)	4 VR sessions—one per week. Participants had to find and crush up to 60 virtual cigarettes.	1. Nicotine dependence	In the experimental group, there was a statistically significant reduction in nicotine addiction, abstinence rate (confirmed with exhaled carbon monoxide), and drop‐out rate from the 12‐week psychosocial minimal‐support treatment programme.
Culbertson et al. (2012)	USA	I	Treatment‐seeking cigarette smokers	11 (5, 6)	42.2 (12.5)	VR‐CET + CBT	Immersion: no Interaction: yes	The placebo‐VR cues sessions	VR‐CET: bi‐weekly 30‐min individual VR sessions before or following group CBT, at least 8 sessions	1. Self‐reports of smoking 2. Exhaled breath CO samples during each CET session 3. Craving ratings	Smoking‐VR CET participants had a higher quit rate than placebo‐VR CET participants. Smoking‐VR CET‐treated participants also reported smoking significantly fewer cigarettes per day at the end of treatment than placebo‐VR CET‐treated participants.
Malbos et al. (2018)	France	I	People with a past diagnosis of chronic smoking—DSM‐5 criteria for nicotine dependence and smoking abstinence of 1 week	61 (n/a)	49 (13)	VR‐CET	Immersion: yes Interaction: yes	CBT	VR‐CET 8 weekly sessions of 45 min; exposure to smoking cues in different situations	1. Tobacco dependence 2. Tobacco craving 3. Anxiety 4. Depression 5. Self‐esteem 6. Presence 7. Tobacco abstinence	Results demonstrated that VR‐CET is at least as efficacious as traditional CBT in terms of maintenance of tobacco abstinence, craving reduction, and decrease in nicotine dependence.
Pericot‐Valverde et al. (2019)	Spain	I	People with a diagnosis of nicotine dependence	102 (50, 52)	39.21 (12.97); 39.68 (12.77)	VR‐CET + CBT	Immersion: yes Interaction: yes	CBT	5 VR‐CET sessions in 6 weeks, VR provided before or after the CBT session, previously validated virtual environments used for eliciting craving.	1. Smoking abstinence 2. Treatment retention 3. Relapse rate 4. Cue‐induced craving	The intervention group showed a significant reduction in cue‐induced craving. There were no significant differences in either retention or abstinence rates between the control and intervention groups.
Goldenhersch et al. (2020)	Argentina	I	People smoking more than 5 cigarettes per day	120 (60, 60)	43.25 (10.06)	VR‐MET	Immersion: yes Interaction: no	The smoking cessation manual	Practice sessions in informal mindfulness using VR included 2 sessions of VR‐MET in a 21‐day programme, each lasting 10 min.	1. Self‐reported abstinence (after a day, after 90 days) 2. Adherence to treatment 3. Cigarette consumption 4. Craving 5. Mindfulness 6. Readiness to quit 7. Nicotine dependence	Participants in the intervention group reported greater abstinence than participants in the control group. Among participants still smoking postintervention, the intervention group was significantly more ready to quit compared with the control group.
Machulska et al. (2021)	Germany	I	People smoking at least 6 cigarettes per day during the last 6 months	96 (47, 49)	50.3 (11.6)	TAU + VR‐ABM	Immersion: yes Interaction: yes	TAU + VR (game)	6 sessions within 2 weeks, approach bias modification VR training—all smoking‐related objects were labelled red and had to be thrown away, whereas non‐smoking‐related objects were grasped/approached.	1. Attitude towards smoking 2. Stages of change 3. Thoughts about abstinence 4. Impulsiveness 5. Positive mental health 6. Alcohol Use Disorders Identification 7. Alcohol use 8. Health behaviour checklist	VR‐ABM did not change approach biases, nor other cognitive biases, but it was superior in reducing daily smoking.
Malbos et al. (2023)	France	I	People with past diagnosis of chronic smoking—DSM‐5 criteria for nicotine dependence and smoking abstinence of 1 week	100 (50, 50)	47.65 (13.31)	VR‐CET	Immersion: yes Interaction: yes	CBT	8 weekly sessions of 45 min; exposure to smoking cues in different situations	1. Tobacco dependence 2. Tobacco craving 3. Anxiety 4. Depression 5. Self‐esteem 6. Quality of life 7. Presence 8. Tobacco abstinence 9. Physiological measures	More participants in the intervention group did not experience smoking relapses. Intervention is at least as efficacious as traditional CBT in terms of craving reduction and decrease in nicotine dependence. Dropout and relapse rates in the intervention group were noticeably lower than in the control group.
Bordnick et al. (2012)	USA	I	Nicotine‐dependent adults	86 (44, 42)	47.9 (10.4), 46.2 (8.4)	NRT + VRST	Immersion: yes Interaction: yes	NRT	VRST combined with NRT for 10 weeks (1 screening and 9 VRST sessions).	1. Cigarette craving 2. Self‐reports of smoking 3. Smoking abstinence self‐efficacy	Smoking rates and craving nicotine were significantly lower for the intervention group compared with the control group at the end of treatment. Self‐confidence and coping skills were also significantly higher for the intervention group, and a number of cigarettes smoked was significantly lower, compared with the control group at follow‐up.
Weser et al. (2021)	USA	P	Middle school students	287 (155, 132)	12.45 (0.57)	VR	Immersion: yes Interaction: yes	TAU	Invite Only VR gameplay to practice refusing peer pressure to vape e‐cigarettes. 2–4 sessions for approximately 40 min	1. General e‐cigarette knowledge 2. Nicotine addiction knowledge 3. Perceived addictiveness of e‐cigarettes 4. Perceptions of e‐cigarette harm 5. Social perceptions about e‐cigarettes	Invite Only VR players improved in e‐cigarette knowledge, nicotine addiction knowledge, perceived addictiveness of e‐cigarettes, perceptions of harm, and social perceptions about e‐cigarette use compared with the control group.
	Illicit drugs										
	Ketamin										
Man (2018)	Hong Kong	I	Ketamine users recruited from the treatment programmes	90 (30, 30 (TAG), 30)	22.80 (5.41); 24.77 (4.14); 24.60 (3.91)	VRVTS	Immersion: no Interaction: yes	Two control groups: 1. delayed VR; 2. TAG (training from a tutor according to a training manual, which was like those experienced by the intervention group).	3 levels with 5 sessions each (15 sessions); 3D nonimmersive type of VR training (virtual reality‐based vocational training system or VRVTS)	1. Cognitive assessment (general intellectual abilities, attention, memory, executive function) 2. Work assessment 3. Participants' knowledge and skills regarding the performance of sales‐related activities 4. Participants' self‐efficacy when performing sales‐related activities	The intervention group exhibited significant improvements in attention and improvements in memory that were maintained after 3 months. Both the intervention and tutor‐administered (control) group exhibited significantly improved vocational skills after training, which were maintained during follow‐up, and improved self‐efficacy.
	Methamphetamine										
Wang et al. (2019)	China	I	Patients with METH dependence	61 (31, 30)	35.03 (7.53); 32.55 (6.64)	TAU + VR	Immersion: yes Interaction: no	TAU	Six VRCP sessions (two times/week) plus TAU (i.e., physical exercises and supportive psychotherapy as usual) during the study period. VR intervention included 8‐min VR video.	1. Heart rate 2. Craving	Patients with METH dependence who received VRCP showed a significantly larger decrease in the score of METH‐craving and METH‐liking from baseline to follow‐up assessments, compared with those who did not receive VRCP. Participants who received VRCP showed a significantly larger decrease in HRV indexes on the time domain and non‐linear domain from baseline to follow‐up assessments during exposure to VR cues, compared with those in a control group.
	Opioids										
Herbert et al. (2020)	USA	P	General population	98 (58, 40)	Median: 40 (E) and 41 (C)	VR video	Immersion: yes Interaction: no	OOPP	One‐time, 9‐min OOPP VR video	1. Opioid overdose knowledge 2. Opioid overdose attitudes	Results demonstrate participants exposed to the immersive video OOPP had equivalent improvements in post‐test knowledge and more favourable attitudes about responding to an opioid overdose than those exposed to the standard OOPP.

Abbreviations: AUD—alcohol use disorder; HRV—heart rate value; ICD‐10—International Classification of Diseases 10th Revision; METH—methamphetamine; N (E—experimental group, C—control group); NRT—nicotine replacement therapy; Objectives (I—intervention, P—prevention); OOPP—opioid overdose prevention programme; TAU—treatment as usual; VAAAT—Virtual Alcohol Approach‐Avoidance Training Task; VR‐ABM—Virtual Reality Approach Bias Modification; VR‐CBT—VR cognitive behavioural therapy; VR‐CET—V cue exposure therapy; VRCP—virtual reality counterconditioning procedure; VR‐MET—VR mindful exposure therapy; VRST—virtual reality skills training; VR—virtual reality; VRVTS—virtual reality–based vocational training system.

^a^ Immersion (i.e., the extent to which participants are immersed in the virtual environment) and interaction (i.e., the degree to which participants can actively engage or manipulate elements within the virtual environment) were assessed based on information provided in the included articles. We categorized the types of immersion according to the display mode: (1) immersive VR environments—virtual environments presented via a head‐mounted display; and (2) nonimmersive VR environments—content displayed on a computer/laptop screen. Interactivity was assessed based on whether participants could interact (e.g., manipulating objects or navigating the scene) with the virtual environment.

In terms of VR technology, we classified the type of immersion based on the display mode: (1) immersive VR environments, defined as virtual environments presented via a HMD; and (2) nonimmersive VR environments, defined as content displayed on a computer or laptop screen (Table 1). The majority of studies (*n* = 16) employed immersive virtual environments presented via a HMD (Hernández‐Serrano et al. 2020; Guldager et al. 2022; Girard et al. 2009; Malbos et al. 2018, 2023; Pericot‐Valverde et al. 2019; Machulska et al. 2021; Bordnick et al. 2012; Weser et al. 2021; Ma 2020; Figueras‐Puigderrajols et al. 2020; Zhang et al. 2023; Thaysen‐Petersen et al. 2024; Goldenhersch et al. 2020; Wang et al. 2019; Herbert et al. 2020). Only four studies used nonimmersive VR environments (Kim and Lee 2019; Gamito et al. 2021; Culbertson et al. 2012; and Man 2018).

The results will be presented according to the substance that the research was focused on: alcohol, nicotine (cigarettes) and illicit drugs.

### 3.1 Alcohol

Of the eight studies focused on alcohol use, six were intervention studies (Figueras‐Puigderrajols et al. 2020; Gamito et al. 2021; Hernández‐Serrano et al. 2020; Kim and Lee 2019; Thaysen‐Petersen et al. 2024; Zhang et al. 2023) and two were prevention studies (Guldager et al. 2022; Ma 2020). Research participants were mostly patients with AUD (Figueras‐Puigderrajols et al. 2020; Gamito et al. 2021; Hernández‐Serrano et al. 2020; Thaysen‐Petersen et al. 2024; Zhang et al. 2023) but also heavy social drinkers (Kim and Lee 2019) and students (Guldager et al. 2022; Ma 2020).

Three studies used VR cue exposure therapy (VR‐CET; Figueras‐Puigderrajols et al. 2020; Hernández‐Serrano et al. 2020; Zhang et al. 2023), two used VR‐assisted CBT sessions (Gamito et al. 2021; Thaysen‐Petersen et al. 2024), two used VR for psychoeducation (Guldager et al. 2022; Ma 2020) and one used Virtual Alcohol Approach‐Avoidance Training Task (VAAAT; Kim and Lee 2019). Study duration varied from one (e.g., Ma 2020) to 10 sessions (Gamito et al. 2021) lasting from 4 to 60 min.

Considering the type of VR experience, six studies implemented immersive VR (Figueras‐Puigderrajols et al. 2020; Hernández‐Serrano et al. 2020; Thaysen‐Petersen et al. 2024; Zhang et al. 2023; Guldager et al. 2022; Ma 2020), i.e., 360° videos, whereas only two studies used nonimmersive VR (Kim and Lee 2019; Gamito et al. 2021).

Different studies measured the effectiveness of VR using different outcomes. All eight studies found significantly higher improvements in the VR conditions for at least one outcome. Specifically, two studies (Figueras‐Puigderrajols et al. 2020; Hernández‐Serrano et al. 2020) evaluated the effectiveness of VR‐CET as an additional treatment to treatment as usual (TAU) on alcohol craving, anxiety and attentional bias. Figueras‐Puigderrajols et al. (2020) found that the decrease in craving, anxiety and attentional bias was more evident in the intervention than in the control group. Hernández‐Serrano et al. (2020) found a greater reduction in the levels of alcohol craving in the intervention than in the control group. Zhang et al. (2023) explored the effects of VR‐based CET on the subjective craving and physiological responses to alcohol cues of patients with alcohol dependence. Results showed that after VR‐CET, the changes in subjective craving and heart rate in the intervention group were significantly lower than in the control group. Gamito et al. (2021) explored whether a cognitive training approach using VR exercises based on activities of daily living is feasible for improving the cognitive function of patients with AUD undergoing residential treatment. Results indicated better cognitive and executive functioning in the experimental group than in the control group (Gamito et al. 2021). Thaysen‐Petersen et al. (2024) investigated the feasibility of using VR‐simulated high‐risk environments in CBT‐based treatment of AUD. Results showed that patients receiving VR‐CBT had more reduction in alcohol consumption and craving than patients receiving conventional CBT at 1‐week and 1‐month follow‐up (Thaysen‐Petersen et al. 2024). Ma (2020) implemented VR immersive stories to prevent college students from drinking and driving. Results showed that an immersive story led to lower intentions to drink and drive only among female participants when viewed through a VR headset (vs. tablets; Ma 2020). Guldager et al. (2022) used VR to improve the refusal self‐efficacy of adolescents who face social pressures to drink alcohol. Results showed no significant difference between the intervention and control groups. Furthermore, Kim and Lee (2019) aimed to reduce the approach tendency towards alcohol among heavy social drinkers using the VAAAT training. The intervention group showed a decrease in implicit approach tendencies towards alcohol but not in explicit craving for alcohol. In contrast, the control group showed an increase in both implicit approach tendencies and explicit craving towards alcohol (Kim and Lee 2019).

### 3.2 Nicotine

Eight studies that examined smoking behaviours were intervention studies (Bordnick et al. 2012; Culbertson et al. 2012; Girard et al. 2009; Goldenhersch et al. 2020; Machulska et al. 2021; Malbos et al. 2018, 2023; Pericot‐Valverde et al. 2019), and one prevention study focused on e‐cigarettes (Weser et al. 2021).

Participants were people with a past diagnosis of chronic smoking (Malbos et al. 2018, 2023), persons with a diagnosis of nicotine dependence (Bordnick et al. 2012; Pericot‐Valverde et al. 2019), cigarette smokers (Culbertson et al. 2012; Girard et al. 2009; Goldenhersch et al. 2020; Machulska et al. 2021) and students (Weser et al. 2021).

In four out of nine studies (Culbertson et al. 2012; Malbos et al. 2018, 2023; Pericot‐Valverde et al. 2019), VR‐CET was used, one study used VR Approach Bias Modification (VR‐ABM; Machulska et al. 2021), one used Virtual Reality Mindful Exposure Therapy (VR‐MET; Goldenhersch et al. 2020), one used Virtual Reality Skills Training (VRST; Bordnick et al. 2012), and two studies used VR gameplay (Girard et al. 2009; Weser et al. 2021). Study duration ranged from 2 (Machulska et al. 2021) to 8 weeks of treatment (Malbos et al. 2018, 2023).

Eight studies used immersive VR (Bordnick et al. 2012; Girard et al. 2009; Goldenhersch et al. 2020; Pericot‐Valverde et al. 2019; Machulska et al. 2021; Malbos et al. 2018, 2023; Weser et al. 2021), and one study used nonimmersive VR (Culbertson et al. 2012).

Six studies measured cigarette craving (Bordnick et al. 2012; Culbertson et al. 2012; Goldenhersch et al. 2020; Malbos et al. 2018, 2023; Pericot‐Valverde et al. 2019), four measured tobacco or nicotine dependence (Girard et al. 2009; Goldenhersch et al. 2020; Malbos et al. 2018, 2023), four measured abstinence from cigarettes (Goldenhersch et al. 2020; Malbos et al. 2018, 2023; Pericot‐Valverde et al. 2019), and three measured self‐reported smoking (Bordnick et al. 2012; Culbertson et al. 2012; Goldenhersch et al. 2020). One study measured knowledge about nicotine addiction and e‐cigarettes, as well as attitudes about e‐cigarettes, behavioural intentions and self‐efficacy to refuse (Weser et al. 2021). Two studies assessed abstinence by measuring expired carbon monoxide (CO) levels (Bordnick et al. 2012; Pericot‐Valverde et al. 2019).

In seven out of nine articles, an effect of VR was found for at least one of the measured outcomes. Specifically, Girard et al. (2009) tested the advantages of combining VR and a psychosocial smoking‐cessation treatment programme for adults. They found that the intervention during four sessions led to a statistically significant reduction in nicotine addiction, abstinence rate and drop‐out rate from the 12‐week psychosocial minimal‐support treatment programme. Culbertson et al. (2012) found that participants in the intervention group had a higher smoking quit rate than participants in the control group, as they smoked significantly fewer cigarettes per day at the end of treatment than those in the control group. In studies of Malbos et al. (2018, 2023), results showed that VR‐CET was at least as efficacious as traditional CBT in terms of maintenance of tobacco abstinence, craving reduction and decrease in nicotine dependence. Pericot‐Valverde et al. (2019) analysed whether adding a VR‐CET protocol to a CBT intervention would increase treatment retention and short‐ and long‐term smoking cessation and found that the intervention group showed a significant reduction in cue‐induced craving, unlike the control group. Goldenhersch et al. (2020) found that participants in the intervention group reported greater abstinence than participants in the control group. Machulska et al. (2021) found that VR intervention did not change approach biases or other cognitive biases, but it was superior in reducing daily smoking. In their study, Bordnick et al. (2012) found that smoking rates and craving for nicotine were significantly lower for the intervention group compared with the control group at the end of treatment. Self‐confidence and coping skills were also significantly higher for the intervention group, and the number of cigarettes smoked was significantly lower compared with the control group at follow‐up (Bordnick et al. 2012). Weser et al. (2021) found that participants who were involved in the VR game improved in e‐cigarette knowledge, nicotine addiction knowledge, perceived addictiveness of e‐cigarettes, perceptions of harm and social perceptions about e‐cigarette use compared with the control group.

### 3.3 Illicit Drugs

In one study, a VR counterconditioning procedure (VRCP; Wang et al. 2019) was used, one used VR vocational training systems (VRVTS), and one study used a VR immersive video based on an opioid overdose prevention programme (OOPP; Herbert et al. 2020). The study duration varied from one‐time sessions (Herbert et al. 2020) to 15 sessions (Man 2018).

Two studies used immersive VR (Wang et al. 2019; Herbert et al. 2020), whereas one study used nonimmersive VR (Man 2018). In two studies (Herbert et al. 2020; Man 2018), intervention group had only a VR intervention, whereas in one study, along with VR, participants had TAU (Wang et al. 2019).

Man (2018) measured cognitive assessment, work assessment, participants' knowledge and skills regarding the performance of sales‐related activities and participants' self‐efficacy when performing sales‐related activities. Wang et al. (2019) measured the heart rate and METH craving, whereas Herbert et al. (2020) wanted to investigate opioid overdose knowledge and opioid overdose attitudes as outcomes.

After VR intervention, results varied in different studies. Man (2018) found that the intervention group exhibited significant improvements in attention and memory maintained after 3 months. The intervention and tutor‐administered (control) group exhibited significantly improved vocational skills after training, which were maintained during follow‐up and improved self‐efficacy. In a study by Wang et al. (2019), patients who received a VRCP showed a significantly larger decrease in heart rate variability (HRV) indexes on the time domain and non‐linear domain from baseline to follow‐up assessments during exposure to VR cues, compared with those in a control group. Herbert et al. (2020) showed that participants exposed to the immersive video based on OOPPs had equivalent improvements in post‐test knowledge and more favourable attitudes about responding to an opioid overdose than those exposed to the standard OOPP.

### 3.4 Risk of Bias in the Included Studies

The risk of bias for the included studies by domain, as well as the overall risk of bias, is presented in Table 2. Although the risk of bias varied across domains among the included studies, most studies (63.2%) showed a high overall risk of bias. A high risk of bias was present across all five domains in different studies; however, it was most prominent in the third domain (missing outcome data), followed by the fourth domain (measurement of the outcome) and the first domain (randomization process). The high risk of bias in the third domain was primarily due to the high attrition and dropout rate of participants through the intervention. Regarding outcome measurement, most of the studies used subjective assessment measures (e.g., craving), with the assessors either not blinded, or the articles did not specify the information needed to determine whether blinding was implemented. Additionally, most articles did not report whether the allocation sequence was concealed prior to participant enrolment and assignment to interventions. Because of this ambiguity of bias in some studies, the first domain was evaluated as high risk, even though proper allocation and randomization procedures may have been implemented; however, this was not reported in the articles. These findings indicate a need for methodological improvements in the aforementioned domains to improve the overall quality of future studies.

**TABLE 2:** Risk of bias assessment.

Study	D1	D2	D3	D4	D5	Overall bias
Kim and Lee (2019)	High risk	Some concerns	Low risk	High risk	Some concerns	High risk
Hernández‐Serrano et al. (2020)	High risk	High risk	High risk	High risk	Some concerns	High risk
Ma (2020)	Some concerns	Low risk	Low risk	High risk	High risk	High risk
Figueras‐Puigderrajols et al. (2020)	Some concerns	Low risk	Low risk	Some concerns	High risk	High risk
Gamito et al. (2021)	Some concerns	Some concerns	Low risk	Some concerns	Low risk	Some concerns
Guldager et al. (2022)	Low risk	Low risk	Low risk	Some concerns	Low risk	Some concerns
Zhang et al. (2023)	Some concerns	High risk	High risk	High risk	Low risk	High risk
Thaysen‐Petersen et al. (2024)	High risk	Some concerns	Low risk	Low risk	Some concerns	High risk
Girard et al. (2009)	Some concerns	Low risk	High risk	Low risk	Some concerns	High risk
Culbertson et al. (2012)	High risk	High risk	High risk	Some concerns	Some concerns	High risk
Malbos et al. (2018)	Some concerns	Low risk	Low risk	Some concerns	Some concerns	Some concerns
Pericot‐Valverde et al. (2019)	Some concerns	Low risk	Some concerns	Low risk	Some concerns	Some concerns
Goldenhersch et al. (2020)	Some concerns	Low risk	High risk	Some concerns	Some concerns	High risk
Machulska et al. (2021)	Some concerns	Low risk	Low risk	Some concerns	Some concerns	Some concerns
Malbos et al. (2023)	Some concerns	Some concerns	High risk	High risk	Some concerns	High risk
Bordnick et al. (2012)	Some concerns	Some concerns	High risk	High risk	Some concerns	High risk
Weser et al. (2021)	High risk	Some concerns	High risk	High risk	Some concerns	High risk
Man (2018)	Low risk	Some concerns	High risk	Low risk	Some concerns	High risk
Wang et al. (2019)	Low risk	Some concerns	Low risk	Low risk	Some concerns	Some concerns
Herbert et al. (2020)	Low risk	Some concerns	Low risk	Some concerns	Some concerns	Some concerns

*Note:* D1—randomization process; D2—deviations from the intended interventions; D3—missing outcome data; D4—measurement of the outcome; D5—selection of the reported result.

## 4 Discussion

This systematic review analysed 20 randomized controlled trials evaluating the effectiveness of VR interventions for preventing and treating substance‐related disorders. The studies encompassed a wide array of VR modalities, highlighting the adaptability and potential of VR in addressing substance‐related disorders. Notably, this is the first systematic review to focus exclusively on the effectiveness of VR in the treatment and prevention of SA, based solely on randomized controlled trials. The review aimed to achieve two main objectives: (1) to detail the various VR interventions employed for substance (ab)use and (2) to assess their effectiveness and the mechanisms that underpin them. By addressing these objectives, the review intended to fill a significant gap in the literature and provide insights into the potential of VR as a therapeutic tool for substance‐related disorders.

### 4.1 Typology of VR Interventions for SUDs

#### 4.1.1 Therapeutic Orientation

The reviewed interventions were based on a variety of therapeutic frameworks. The most frequently applied orientation was CET. Eight studies used classical VR‐CET protocols involving repeated exposure to substance‐related cues with response prevention (e.g., Culbertson et al. 2012; Malbos et al. 2023; Thaysen‐Petersen et al. 2024). Two studies employed modified exposure‐based approaches in which cue exposure was embedded within distinct therapeutic paradigms. In one study, participants were guided to observe smoking‐related cues mindfully, with an emphasis on present‐moment awareness and emotional acceptance rather than response prevention or habituation (Goldenhersch et al. 2020). In the other, exposure to alcohol cues was combined with avoidance‐oriented motor responses in a gamified task designed to retrain automatic approach tendencies (Kim and Lee 2019). CBT was used in five studies, either as VR‐assisted CBT (e.g., Thaysen‐Petersen et al. 2024) or as a standard CBT programme combined with VR treatments (e.g., Culbertson et al. 2012).

In addition to CBT and CET, several studies employed other therapeutic orientations. Approach bias modification (ABM) was the core strategy in two studies, using VR to retrain automatic approach tendencies towards substance cues (Kim and Lee 2019; Machulska et al. 2021). Skills training interventions were implemented in two studies: one aimed at vocational and cognitive rehabilitation for ketamine users (Man 2018), and another combined skills rehearsal with CBT and CET elements to enhance coping in high‐risk situations (Bordnick et al. 2012). One study targeted cognitive rehabilitation, using VR‐based tasks to improve attention and executive function in individuals with AUD (Gamito et al. 2021). A counterconditioning paradigm was applied to modify affective responses to methamphetamine cues (Wang et al. 2019). Finally, psychoeducational and prevention‐oriented interventions, often delivered through immersive video or gamified simulations, were employed to promote knowledge, refusal skills or attitudinal change (e.g., Girard et al. 2009; Guldager et al. 2022; Weser et al. 2021).

Several studies combined more than one therapeutic strategy. For example, Bordnick et al. (2012) integrated CBT and CET elements within a VR‐based skills training protocol, and Culbertson et al. (2012) combined traditional CBT with VR‐CET for a smoking cessation programme. These examples highlight the flexibility of VR as a delivery platform capable of supporting multicomponent therapeutic interventions.

#### 4.1.2 Degree of Immersion

The reviewed studies deployed VR on a continuum ranging from flat‐screen desktop simulations to HMDs. Four studies (Kim and Lee 2019; Gamito et al. 2021; Man 2018; Culbertson et al. 2012) relied on nonimmersive systems in which participants interacted with a scene on a monitor using conventional input devices. In these studies, therapeutic targets were cognitive functions (e.g., attention and memory) or vocational skills rather as well craving. Sixteen studies adopted immersive solutions (e.g., Ma 2020; Pericot‐Valverde et al. 2019; Hernández‐Serrano et al. 2020), which were dominantly used in studies employing CET for alcohol and nicotine, and the majority reported clinically meaningful reductions in craving or substance use (e.g., Hernández‐Serrano et al. 2020). These findings demonstrate that immersive systems provide adequate sensory isolation to induce cue‐reactivity while maintaining feasibility and cost‐effectiveness in outpatient clinical settings. Immersive VR was also used in prevention studies (e.g., Guldager et al. 2022) and for multicomponent smoking programmes (e.g., Bordnick et al. 2012). In sum, although deeper immersion intuitively enhances presence, its actual therapeutic value in addiction interventions remains uncertain and warrants systematic direct comparisons across immersion levels.

A notable limitation across the included studies was the insufficient assessment of participants' sense of presence and prior experience with VR, both of which may influence treatment outcomes by enhancing novelty effects and shaping expectations (Colombo et al. 2021). On the one hand, prior VR experience may impact intervention results, as initial curiosity or novelty effects can diminish with repeated exposure. This is particularly relevant in interventions requiring multiple sessions; for example, in a VR‐based study on autobiographical memory, emotional responses induced by VR significantly decreased as early as the second session (Fernandez‐Alvarez et al. 2021). On the other hand, the sense of presence—defined as the subjective feeling of ‘being there’ within the virtual environment (Riva 2009)—may also contribute to treatment efficacy. However, findings in the literature remain mixed, with some studies suggesting that a greater presence enhances VR effectiveness (Triberti et al. 2025), whereas others report no significant association with treatment outcomes, particularly in VR exposure therapy (Price and Anderson 2007). Among the reviewed studies, only one assessed participants' prior VR experience and found no significant effect on primary outcomes (Malbos et al. 2023). In contrast, four studies assessed the sense of presence as a proxy for immersion and perceived realism (Malbos et al. 2018; Malbos et al. 2023; Weser et al. 2021; Girard et al. 2009). Notably, only two of these studies incorporated presence as an analytical variable, and both found that a higher sense of presence significantly predicted more favourable treatment outcomes (Malbos et al. 2023; Girard et al. 2009). These findings highlight the need for further research to disentangle the extent to which observed improvements are attributable to factors such as presence and novelty, rather than the specific therapeutic mechanisms of the intervention.

#### 4.1.3 Level of Interactivity

VR interventions also differed in their level of interactivity. Some were passive, such as noninteractive 360° videos presented via HMDs, where participants could observe the environment but could not influence the scenario or engage with virtual elements (e.g., Herbert et al. 2020; Ma 2020). Other experiences allowed for more participant engagement, enabling users to navigate environments or make limited scenario‐based choices (e.g., Girard et al. 2009; Guldager et al. 2022).

Some interventions encouraged interaction between participants and the simulation using avatars, which can enhance the sense of presence in VR environments (Hernández‐Serrano et al. 2020). However, these studies often relied on stylized designs and simplified graphics, which may reduce ecological validity (e.g., Hernández‐Serrano et al. 2020; Machulska et al. 2021; Malbos et al. 2018, 2023). In contrast, other interventions were designed as pre‐recorded scenarios with professional actors and real‐world settings, such as bars or restaurants, offering high visual realism but limited interactivity (e.g., Herbert et al. 2020; Thaysen‐Petersen et al. 2024; Wang et al. 2019). This trade‐off between interactivity and realism is an important consideration in designing VR protocols for substance use interventions. Notably, this variation in interactivity was accompanied by marked heterogeneity in intervention length and intensity. Some interventions consisted of a single session lasting under 10 min (e.g., Herbert et al. 2020), whereas others involved structured programmes spanning multiple weeks, with repeated sessions ranging from 30 to 60 min (e.g., Gamito et al. 2021; Malbos et al. 2023). These differences often reflect the complexity of therapeutic goals: Although attitude change or knowledge acquisition may require brief interventions (e.g., Herbert et al. 2020; Weser et al. 2021), cognitive restructuring, skill acquisition or craving desensitization typically necessitates repeated and interactive formats (e.g., Gamito et al. 2021; Thaysen‐Petersen et al. 2024; Zhang et al. 2023).

#### 4.1.4 Psychological Processes Targeted by the Interventions

Across studies, interventions targeted a range of mechanisms associated with SUD. Craving was among the most frequently targeted processes, particularly in studies employing VR‐CET (e.g., Culbertson et al. 2012; Wang et al. 2019). Other interventions sought to reduce implicit cognitive biases such as approach tendencies towards substance‐related cues (e.g., Machulska et al. 2021) or to improve executive functioning and attention (e.g., Gamito et al. 2021; Man 2018). Psychoeducational and narrative VR interventions primarily targeted knowledge acquisition, attitudinal shifts and behavioural intentions (e.g., Ma 2020; Weser et al. 2021), whereas skill‐based simulations focused on increasing self‐efficacy and coping capacity (e.g., Bordnick et al. 2012).

### 4.2 Effectiveness of VR Interventions

Across included studies, evidence for the effectiveness of VR interventions in SA treatment and prevention is mixed but largely supportive. In total, in 17 out of 20 studies, a positive effect was found in at least one outcome variable. However, there was variation in both the nature of outcomes and the strength of supporting evidence. Specifically, outcomes can be broadly categorized into clinically meaningful outcomes, such as a reduction in substance use (e.g., decreased frequency or quantity of use), point‐prevalence abstinence (e.g., quit rates assessed at the end of treatment) and relapse, and proximal or indirect indicators, including craving, physiological responses, cognitive improvements and attitudinal and knowledge changes.

Clinically meaningful outcomes were assessed in 10 of the 20 included studies, most commonly focusing on reduction in substance use, followed by quit rates and relapse. Among these, seven studies demonstrated significant improvements in at least one of these outcomes in the VR condition. Reductions in use, typically operationalized as decreased frequency or quantity of consumption, were reported in studies targeting both smoking (e.g., Culbertson et al. 2012) and alcohol (e.g., Thaysen‐Petersen et al. 2024). Abstinence was measured in only four studies, and in two of these (Girard et al. 2009; Goldenhersch et al. 2020), VR interventions outperformed control conditions. Importantly, both studies targeted smoking cessation and employed short‐term, point‐prevalence abstinence measures (e.g., quit rates at post‐treatment), limiting conclusions about sustained change. This points to an important gap in literature—abstinence remains underassessed, and where assessed, it is limited to nicotine‐related interventions. To strengthen the clinical relevance of VR‐based interventions, future research should systematically incorporate abstinence as a primary outcome, particularly for substances beyond nicotine, and assess it using longer‐term follow‐ups.

In contrast, proximal and intermediate outcomes were assessed in most of the studies (19 out of 20). Craving was the most frequently measured outcome, reported in 11 studies. Other categories of proximal outcomes included attitudinal or knowledge‐related outcomes, such as intentions to use substances, beliefs about harm, refusal self‐efficacy and awareness of risks (e.g., Guldager et al. 2022; Ma 2020; Weser et al. 2021); cognitive outcomes, including attention, memory, executive functioning and approach or avoidance tendencies towards substance cues (e.g., Gamito et al. 2021; Kim and Lee 2019; Man 2018); and physiological and affective responses, such as heart rate and state anxiety (e.g., Figueras‐Puigderrajols et al. 2020; Zhang et al. 2023). Across these domains, 13 studies found positive effects of VR interventions on at least one proximal outcome. These findings underscore the multifaceted impact of VR interventions on various aspects of SUDs.

Importantly, several studies reported null findings. For example, VR interventions targeting alcohol refusal self‐efficacy (Guldager et al. 2022), explicit alcohol craving (Figueras‐Puigderrajols et al. 2020; Kim and Lee 2019), smoking abstinence (Pericot‐Valverde et al. 2019) and smoking approach bias (Machulska et al. 2021) showed no significant effects. One study even found a higher relapse rate in the VR intervention group for smoking cessation at 12‐month follow‐up (Pericot‐Valverde et al. 2019). Different factors may contribute to these null findings. Some VR interventions may have lacked sufficient engagement or immersion because of factors such as poor virtual environment design, conducting only a single session (e.g., Guldager et al. 2022), or a mismatch between the virtual experience and participants' individual needs and preferences. The virtual environments in several studies may not have adequately represented real‐world situations and triggers, limiting their ecological validity. Additionally, some studies suffered from low statistical power because of relatively small sample sizes (e.g., Figueras‐Puigderrajols et al. 2020), making detecting even moderate treatment effects difficult. Furthermore, uncontrolled extraneous variables (e.g., differences in comorbidity or medication adherence in Figueras‐Puigderrajols et al. 2020) may have confounded the results. A ceiling effect in control groups (e.g., the high abstinence rates in the CBT‐only group in Pericot‐Valverde et al. 2019) might also have obscured any additional benefits of the VR intervention.

In sum, evidence for proximal outcomes appears more consistent and robust across studies, whereas findings related to clinically meaningful outcomes such as abstinence and relapse are more variable and limited, highlighting the need for more rigorous trials with long‐term follow‐up.

### 4.3 Working Mechanisms

The effectiveness of VR interventions used in the studies included in this systematic review is derived from various mechanisms grounded in different theoretical frameworks, as well as the inherent qualities of VR. For example, interventions like the VAAAT (e.g., Kim and Lee 2019) utilize a counter‐conditioning approach. This method involves repeatedly pairing alcohol‐related virtual stimuli with avoidance responses, while associating non‐alcohol–related stimuli with approach responses. The goal is to weaken the link between alcohol cues and approach behaviour, ultimately replacing it with an association geared towards avoidance.

Other studies utilized principles of CET (e.g., Culbertson et al. 2012; Hernández‐Serrano et al. 2020) by exposing participants to virtual cues strongly linked to substance cravings—such as bottles, cigarettes, drinks, bars and restaurants—during extended sessions where they were unable to ‘virtually’ drink or smoke (response prevention). This method is believed to facilitate systematic desensitization to substance‐related cues and contexts, ultimately reducing cravings. Additionally, VR enhances the effectiveness of CET by incorporating multiple sensory inputs, including visual, auditory and olfactory stimuli. This greater sensory engagement allows clinicians to maintain better control over the exposure process (Ferrer‐Garcia et al. 2019). Furthermore, integrating mindfulness practices into VR‐CET may offer a novel mechanism of action. By promoting awareness of thoughts, feelings and sensations related to cravings, mindfulness can facilitate self‐acceptance and emotional regulation, potentially augmenting treatment outcomes (Goldenhersch et al. 2020). It is important to note that CET is considered a component of a broader CBT approach for treating SUDs (Thaysen‐Petersen et al. 2024). Some studies also used other CBT techniques such as cognitive restructuring in which patients needed to identify and challenge their maladaptive thoughts and beliefs related to substance use and high‐risk situations or learn and practise coping strategies (e.g., distraction and breathing exercises) to manage cravings and resist high‐risk behaviours (Thaysen‐Petersen et al. 2024). Moreover, under the CBT framework, Gamito et al. (2021) implemented cognitive retraining through VR‐based exercises that simulate daily living activities, allowing participants to enhance their cognitive skills. Another mechanism highlighted in studies is immersive storytelling within VR (e.g., Ma 2020), which can enhance the story's persuasiveness and influence psychological processes, ultimately helping to prevent risky behaviours. VR interventions incorporating game‐like activities may have yielded positive results by enhancing skill learning, participants' self‐efficacy (belief in their ability to quit) and promoting conditioned learning, where the automatic urge to use the substance is replaced with an alternative action (e.g., Girard et al. 2009).

The question arises regarding the effectiveness of VR interventions in comparison with traditional therapeutic approaches. As already discussed, the studies included in this systematic review primarily utilized VR to deliver therapeutic modalities developed within the framework of CBT. The most frequently applied methods were CET, various other CBT techniques or a combination of CET with additional CBT approaches. Consequently, it is important to consider whether the incorporation of a VR environment as a medium for delivering these interventions enhances their value compared with traditional, technology‐free interventions. However, only a limited number of included studies permitted a discussion of this topic because of their specific designs. For instance, Malbos et al. (2018) demonstrated that VR‐CET, when combined with non‐VR CBT techniques, was at least as effective as traditional CBT in promoting the maintenance of tobacco abstinence, reducing cravings and decreasing nicotine dependence. Culbertson et al. (2012) reported that smoking participants who underwent VR‐CET in conjunction with traditional CBT achieved a higher quit rate and smoked fewer cigarettes per day by the end of treatment compared with those who received a placebo‐VR‐CET combined with traditional CBT. Likewise, Pericot‐Valverde et al. (2019) found that the VR‐CET + CBT group experienced a significant reduction in cue‐induced smoking cravings compared with the group that received conventional CBT. However, it is noteworthy that no significant differences were observed in retention or abstinence rates between the groups. Thaysen‐Petersen et al. (2024) found that AUD patients receiving VR‐CBT had more reduction in alcohol consumption and craving than patients receiving conventional CBT at 1‐week and 1‐month follow‐up. Although limited, these findings are promising, suggesting that VR technology can offer incremental benefits to already established and widely used therapeutic strategies. Taken together, the findings suggest that VR serves not merely as a vehicle for delivering existing therapeutic modalities but as an active enhancer of their core mechanisms. Through rich sensory stimulation (e.g., visual, auditory and olfactory), increased therapist control over stimulus presentation and the ability to simulate complex, ecologically valid scenarios, including emotionally salient and socially embedded situations, VR can strengthen key therapeutic processes such as presence, emotional engagement, experiential learning and behavioural rehearsal (Hernández‐Serrano et al. 2020). These features may enhance a range of mechanisms commonly used in substance use treatment, including cue exposure, cognitive restructuring, mindfulness and skill acquisition. The heightened sense of presence, often described as the subjective experience of ‘being there’ (Simon et al. 2020), enables individuals to engage with therapeutic content more deeply, respond to virtual cues as if they were real and practice adaptive strategies in safe but realistic contexts. As a result, individuals may be better able to generalize therapeutic gains and implement coping strategies more effectively in everyday situations associated with substance use (Ghiță and Gutiérrez‐Maldonado 2018).

### 4.4 Limitations

This study has several limitations that need to be addressed. First is the heterogeneity of the included studies, which is evident in the types of VR interventions employed; the specific substance use that was addressed; the duration of these interventions; the outcome variables; and the way they were measured. This poses a challenge in drawing definitive conclusions regarding the effectiveness of VR in treating and preventing substance‐related behaviours. Moreover, the quality of this review is directly influenced by the methodological rigour of the included studies. Despite our efforts to prioritize studies with robust scientific methodologies by limiting our selection to RCTs, certain shortcomings remain. For instance, several studies featured small sample sizes, which may have diminished their statistical power to identify significant differences. Several studies did not control for potential confounders (e.g., comorbid conditions) that could influence treatment outcomes. Additionally, the absence of follow‐up measures in many studies restricts our ability to assess the long‐term effects of VR interventions. Publication bias also presents a potential concern, as studies demonstrating significant results are often more likely to be published compared with those with non‐significant or negative findings. This is a concern especially when studies have small sample sizes and low statistical power, as the significant findings may then be due to pure chance, even if the corresponding *p*‐values are low (Joober et al. 2012). The results of the risk of bias assessment highlight limitations due to the methodological quality of the included studies, as most had a high overall risk, primarily because of missing outcome data, subjective outcome measures without proper blinding and unclear randomization procedures. Very few of the included studies reported computing statistical power to detect significant effects (Figueras‐Puigderrajlos et al. 2020; Guldager et al. 2022; Herbert et al. 2020), and sample sizes were usually small. The authors did often note in Section 4 that findings need to be replicated in larger samples. An additional concern is that, in the studies included in this systematic review, many outcomes were routinely tested within a single study and only two studies reported using a correction for multiple testing (Wang et al. 2019; Gamito et al. 2021). The *p* < 0.05 significance criterion rests on the assumption that only one statistical test will be conducted on the data. Conducting multiple tests can substantially increase the odds that spurious findings will be treated as significant. A further remedy is the preregistration of the study and the analyses used (Banks et al. 2018), but that was rarely, if ever, implemented. Although many studies did include active control conditions, as previously mentioned, more studies are needed that would permit direct comparison of VR to traditional treatment approaches to determine its added value. Moreover, this review is limited to research published in English, potentially excluding VR‐related studies released in other languages.

### 4.5 Implications and Future Directions

This systematic review highlighted the significant potential of VR interventions in addressing SA. The findings indicated that VR can influence various key aspects of substance‐related disorders. Specifically, VR interventions demonstrated effectiveness in reducing addiction symptoms such as cravings, enhancing abstinence rates and decreasing the frequency of substance use, particularly for alcohol and nicotine dependence. The primary mechanisms driving these improvements include exposure therapy and CBT techniques. Additionally, this review suggests that VR can be utilized for cognitive training, skills development (e.g., refusal skills) and fostering healthier attitudes and beliefs regarding substance use, underscoring its capacity as a useful tool for behavioural change. The immersive and interactive qualities of VR can also enhance patient motivation and treatment adherence. Moreover, delivering traditional therapies, such as CBT, in a VR environment may offer incremental benefits in treatment outcomes. However, it is important to acknowledge that research in this area remains limited. Finally, VR interventions tend to be brief and require fewer sessions (e.g., eight sessions lasting 30 min each), making them a cost‐effective treatment option.

Although VR interventions tend to be brief (e.g., eight sessions lasting 30 min each), making them a cost‐effective treatment option, research in this area remains limited. A persistent limitation highlighted in this systematic review is the restricted accessibility of validated VR environments from previous studies. Enhancing transparency and promoting open sharing of these resources would substantially benefit future research by enabling greater comparability across studies, supporting the adoption of best practices and accelerating the development and dissemination of effective VR‐based interventions. Furthermore, this open‐access approach could play a critical role in bridging the gap between empirical findings and their application in clinical and educational settings. In this context, a key challenge is the development of mobile‐based VR applications that can be seamlessly integrated into individuals' daily routines through affordable HMDs, such as Google Cardboard. Although this specific application has yet to be empirically investigated in the field of SUDs, the broader potential of VR‐based mobile applications is supported by an emerging body of evidence demonstrating its efficacy in the treatment of anxiety disorders and specific phobias (e.g., Donker et al. 2019; Kim et al. 2017; Miloff et al. 2019).

Besides, the diversity of VR interventions among the studies emphasizes the need for better standardization in terms of treatment manuals, outcome measures and hardware standards. The development of specific guidelines would provide essential support for both researchers and clinicians in designing and implementing VR interventions for the treatment of SUDs. Such standardization would facilitate more robust comparisons across studies, improve research quality and help identify which patient populations may benefit more from VR‐based interventions compared with traditional approaches. Although this review identified VR as a promising treatment modality for substance use, further research is necessary to directly compare the effectiveness of VR interventions with traditional treatment approaches. Additionally, more longitudinal studies are needed to assess the long‐term effects of VR interventions on outcomes such as relapse rates and quality of life. Future studies should also examine the long‐term cost‐effectiveness of VR treatments in comparison with conventional therapies. Given that the most used therapeutic modality within VR has been exposure therapy and other CBT techniques, there is a need to examine the functionality of other therapeutic modalities in VR. For example, in recent years, contextual CBTs, such as Acceptance and Commitment Therapy (ACT), Dialectical Behaviour Therapy (DBT) and mindfulness‐based relapse prevention, have been utilized to treat SA (Lee et al. 2015). Therefore, it would be important to explore the possibility of adapting these therapies to a VR environment. These therapies emphasize present‐moment awareness, emotional regulation, values‐based action and experiential learning—core processes that align well with the features of VR. For instance, preliminary findings suggest that VR may enhance engagement with mindfulness exercises by offering immersive, focused environments that reduce external distractions (Navarro‐Haro et al. 2019). Similarly, VR can provide emotionally engaging, realistic contexts that support key ACT processes such as acceptance, cognitive defusion and values‐based action in an embodied and interactive way. As these processes are often abstract and experientially oriented, VR may help make them more tangible by embedding them in vivid, metaphor‐driven scenarios. Supporting this, Gorinelli et al. (2023) demonstrated that ACT exercises delivered within virtual social situations improved psychological flexibility and reduced anxiety symptoms. VR may also complement DBT, which emphasizes skill development in areas such as distress tolerance, emotion regulation and interpersonal effectiveness. These skills are typically practiced in emotionally evocative situations—something VR can simulate safely and repeatably. By recreating challenging interpersonal or affective contexts, VR enables individuals to rehearse DBT strategies (e.g., urge surfing and opposite action) in lifelike scenarios, potentially increasing emotional engagement, skill acquisition and generalization (Lamb et al. 2023).

Future studies should prioritize reducing attrition by implementing strategies to enhance participant retention and clearly reporting how missing data are handled. Moreover, the use of objective outcome measures and blinding of assessors is essential to minimize measurement bias. Detailed reporting of the randomization process is also crucial to improve transparency and allow for accurate risk of bias assessment, underscoring the need for more rigorous reporting standards in future research.

Although this review identified several mechanisms through which VR interventions may operate, a deeper understanding of these mechanisms and their interaction with individual patient differences is needed. Furthermore, we found limited evidence regarding the application of VR for illicit drug use, indicating a need for more research to adapt and evaluate VR interventions for these specific populations.

## Data Availability

The data that support the findings of this study are available from the corresponding author upon reasonable request.
